# Identification and functional characterization of two bamboo *FD* gene homologs having contrasting effects on shoot growth and flowering

**DOI:** 10.1038/s41598-021-87491-6

**Published:** 2021-04-12

**Authors:** Smritikana Dutta, Anwesha Deb, Prasun Biswas, Sukanya Chakraborty, Suman Guha, Devrani Mitra, Birgit Geist, Anton R. Schäffner, Malay Das

**Affiliations:** 1 Department of Life Sciences, Presidency University, Kolkata, India; 2 Department of Botany, Kalna College, Kalna, West Bengal India; 3 Department of Statistics, Presidency University, Kolkata, India; 4 Institute of Biochemical Plant Pathology, Department of Environmental Sciences, Helmholtz Zentrum München, München, Germany

**Keywords:** Evolution, Molecular biology, Plant sciences

## Abstract

Bamboos, member of the family Poaceae, represent many interesting features with respect to their fast and extended vegetative growth, unusual, yet divergent flowering time across species, and impact of sudden, large scale flowering on forest ecology. However, not many studies have been conducted at the molecular level to characterize important genes that regulate vegetative and flowering habit in bamboo. In this study, two bamboo *FD* genes, *BtFD1* and *BtFD2,* which are members of the florigen activation complex (FAC) have been identified by sequence and phylogenetic analyses. Sequence comparisons identified one important amino acid, which was located in the DNA-binding basic region and was altered between *BtFD1* and *BtFD2* (Ala146 of BtFD1 *vs.* Leu100 of BtFD2). Electrophoretic mobility shift assay revealed that this alteration had resulted into ten times higher binding efficiency of BtFD1 than BtFD2 to its target ACGT motif present at the promoter of the *APETALA1* gene. Expression analyses in different tissues and seasons indicated the involvement of *BtFD1* in flower and vegetative development, while *BtFD2* was very lowly expressed throughout all the tissues and conditions studied. Finally, a tenfold increase of the *AtAP1* transcript level by *p35S::BtFD1 Arabidopsis* plants compared to wild type confirms a positively regulatory role of *BtFD1* towards flowering. However, constitutive expression of *BtFD1* had led to dwarfisms and apparent reduction in the length of flowering stalk and numbers of flowers/plant, whereas no visible phenotype was observed for *BtFD2* overexpression. This signifies that timely expression of *BtFD1* may be critical to perform its programmed developmental role *in planta*.

## Introduction

Bamboos belong to the subfamily Bambusoideae, family Poaceae and are widely distributed in Asia, Africa and America^1,2^. The plant group displays a wide range of variation across species with respect to flowering time and nature. Here flowering takes place after a prolonged vegetative phase, which may be extend up to 120 years^3^. When flowering occurs in a few culms of a population it is called sporadic flowering^4^, while in gregarious flowering a long stretch of geographical area is influenced for blooming^5^. Usually bamboo flowering is followed by death of each and individual culm and is known as monocarpy or semelparity.

Onset of flowering under favourable environment is decided by a complex regulatory crosstalk at molecular level and several mechanisms such as photoperiod, vernalization, autonomous, hormonal and age pathways have been characterized in plants^6–10^. In silico studies indicate that the majority of these pathways also exist in bamboo^11^. Non targeted transcriptome sequencing has been undertaken to identify floral tissue specifically expressed sequence tags (ESTs) of short lengths from many temperate/tropical, woody/herbaceous bamboo species such as *Dendrocalamus* *latiflorus*^12,13^, *Phyllostachys edulis*^14–16^, *P. violascens*^17^, *P*. *aurea, Guadua inermis, Otatea acuminata, Lithachne pauciflora*^18^ and *Fargesia macclureana*^19^. In addition, identification and expression analyses of a group of floral pathway genes or gene families have been undertaken. For example, ten genes related to floral transition and meristem identity were identified in *D. latiflorus*^20^, whereas sixteen MADS box genes were reported from *Bambusa edulis*^21^. A few studies have been conducted to functionally characterize important flowering genes such as *MADS18* from *D. latiflorus*^22^, *FLOWERING LOCUS T* (*FT*) from *P. meyeri*^23,24^, *TERMINAL FLOWER 1* (*TFL1)* like gene from *B. oldhamii*^25^, *FRIGIDA* (*FRI)* from *P. violascens*^26^, and *MADS1, 2* from *P. praecox*^27^.

*FD* genes encoding transcription factors are members of the group A basic leucine zipper (bZIP) family^28^. They are ubiquitously found in angiosperms, but not in any other plant lineages^29^. Studies conducted on *Arabidopsis* (*A. thaliana*) and rice (*O. sativa*) suggest that the transition of shoot apical meristem (SAM) to inflorescence meristem (IM) is primarily governed by interaction among AtFT/OsHd3a, At14-3–3/OsGf14 and AtFD/OsFD1 proteins to form the florigen activation complex (FAC) preceding flowering^30–34^. Subsequently, FD binds to the CRE binding element (ACGT) present in the promoter of floral meristem identity gene *APETALA1*^33,35,36^ (*AP1*). Two paralogous copies of *FD* genes (*AtFD* and *AtFDP*) have been identified in *Arabidopsis*^37^, whereas three copies are present in rice^29^. Other than these reference plants, *FD* homologs have been discovered from many other plants.

The loss-of-function mutation of either *AtFD* or *AtFDP* resulted in late flowering in *Arabidopsis*, while their overexpression demonstrated early flowering indicating their possible involvement in flowering^37,38^. Similarly, the RNAi lines of *OsFD1* demonstrated a late flowering phenotype, while overexpression of *OsFD1* resulted into early flowering^29,33^. In addition to flower induction, other pleiotropic roles of *FD* genes such as inflorescence development^39,40^, leaf development^29^ and alternative growth cessation^41,42^ have also been observed. This clearly indicates that *FD* performs diverse important roles in the vegetative and reproductive developments of plants.

Therefore, in order to understand the diverse functions of *FD* genes in plant growth and development, new studies need to be conducted on yet unexplored, non-reference plants demonstrating remarkable vegetative or flowering habit. Bamboos represent a particularly interesting plant group due to their semelparous life cycle and transition to flowering after decades of vegetative growth. Therefore, the main aim was to identify and characterize bamboo *FD* genes. This study addressed the sequence diversity and differential DNA binding properties of two *FD* genes isolated from *Bambusa tulda* in conjunction with their functional diversity based on expression patterns and impact on vegetative and flowering development in a heterologous system.

## Results

### Identification and sequence characterization of *BtFD1* and *BtFD2* genes

**Figure 1:**
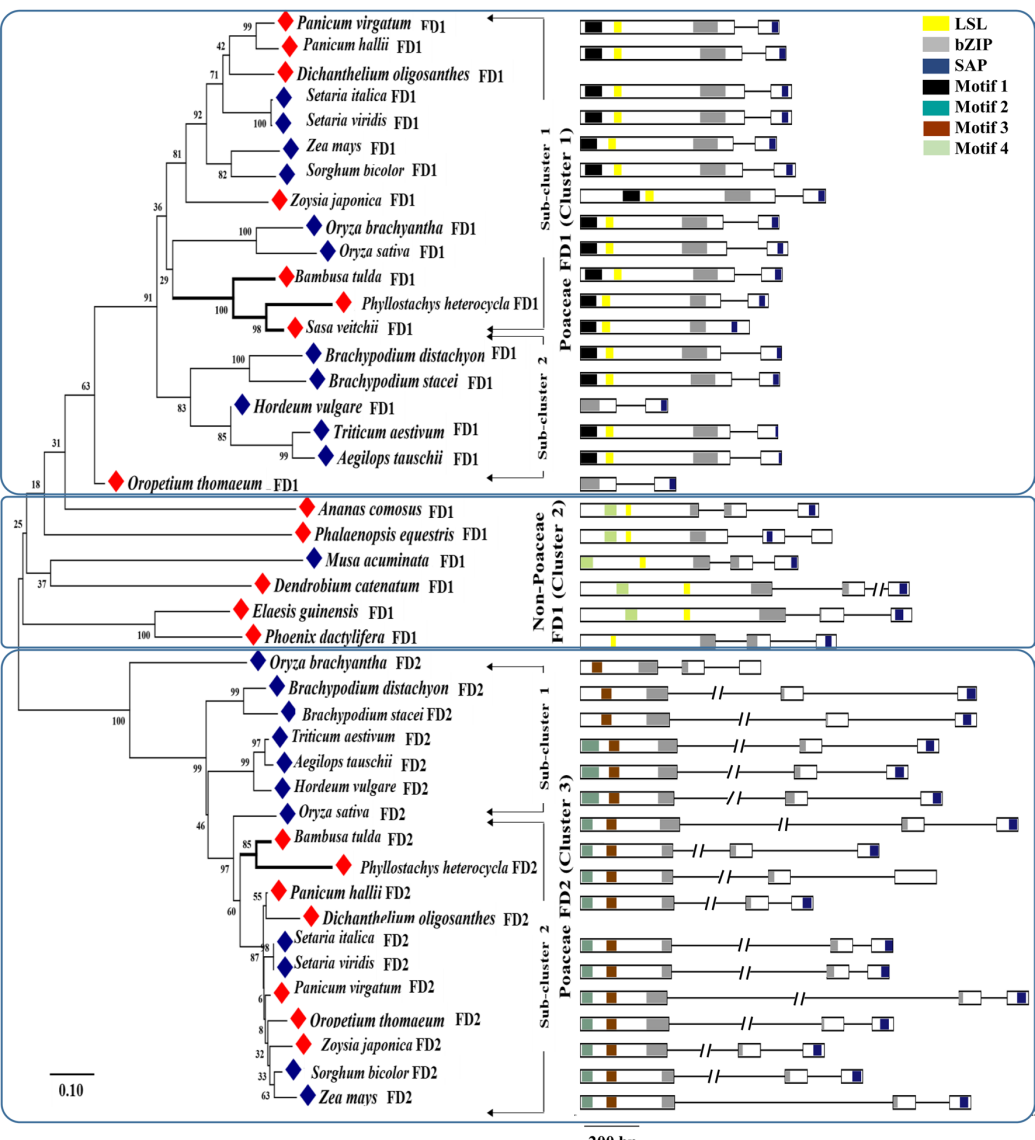
Phylogenetic analysis and predicted gene models of *FD* homologs identified in monocotyledonous plants. The Neighbour Joining (NJ) tree of the *FD* homologs was constructed with the full-length protein sequences by Mega 7.0 using default parameters and bootstrap value 2000. In the gene structures exons were marked as rectangles having conserved motifs marked in solid boxes and introns as solid lines. Annual species were marked in blue and perennials in red rhomboids. The motifs conserved are M 1 [MEDD(E/D)DMW(A/G)XTSSPSASPP], M 2 [NYHHYQMAV(A/H)AA], M 3 [(L/M/V)SGCSSLFSIS(S/T)], M 4 [(M/V)EEVWKDINLSSLHD], LSL [T(A/V)LSLN], Bzip and SAP [(S/T)LXRX(S/T)(A/T)(P/Q)F].

To study the role and diversity of *FD* genes (Table 1) in bamboo, *B. tulda* was selected, because its floral developmental stages have relatively been better characterized than any other bamboo species^11,43^, occurrence of sporadic flowering events in the species from time to time^4,44^ and its enormous economic importance in Asia. Two copies of *BtFD* genes have been identified by designing primers from the conserved regions of homologous FD genes, PCR and multiple sequencing (Supplementary Fig. S1, Fig. 1, *BtFD1*: *MF983712* and *BtFD2: MH142577*). Homology search of the *BtFD1* sequence identified *Sasa veitchii* (Bambusoideae) *FD* (*SvFDL1*: *BAS04368.1*, *SvFDL2*: *BAS04369.1*) as its closest homolog having highest similarity (77%), while the *BtFD2* sequence revealed highest similarity (92%) against *Panicum hallii FD* homolog (*XP_025812603.1*). Predicted lengths of *BtFD1* and *BtFD2* proteins were 202 and 159 amino acids, respectively, and both of them contain the characteristic *bZIP* domain. However, differences between BtFD1 and BtFD2 proteins were observed with reference to other domains. *BtFD1* contains motif 1 [MEDD(E/D)DMW(A/G)XTSSPSASPP], the LSL motif [T(A/V)LSLN] and the SAP motif [(S/T)LXRX(S/T)(A/T)(P/Q)F], while BtFD2 contains motif 2 [NYHHYQMAV(A/H)AA], motif 3 [(L/M/V)SGCSSLFSIS(S/T)] and a modified SAP motif (Supplementary Fig. S2). A detailed sequence comparison of the bZIP domains present in BtFD1 and BtFD2 proteins identified five amino acids changes (Supplementary Fig. S3). Out of these, only the change of Ala146 (BtFD1) > Leu100 (BtFD2) was located in the DNA binding basic region. Therefore, it was investigated whether this amino acid change may or may not impact the binding efficiency of BtFD1 and BtFD2.

**Table 1:** In silico identification of *FD* gene homologs identified from the Poaceae and non-Poaceae members of monocotyledonous plants.

Monocot plant groups	Plant species	Flowering habit	*FD1* homologs identified				*FD2* homologs identified			
			Locus ID/ Accession number of best BLAST hit	Identity (%)	Query cover (%)	E value	Locus ID/ Accession number of best BLAST hit	Identity (%)	Query cover (%)	E value
Poaceae	*Oryza sativa*	Annual	OS09G36910	100	100	0.0	OS06G50830	100	100	0.0
	*O. brachyantha*	Annual	OB09G24570	74	76	2^e-72^	OB02G45490	76	41	4^e-25^
	*Zea mays*	Annual	ZM00001D022613	45	55	9^e-37^	ZM00001D036392	76	80	1^e-74^
	*Hordeum vulgare*	Annual	HVU0041G3471	70	45	3^e-18^	HVU0045G2520	74	80	1^e-74^
	*Sorghum bicolor*	Annual	SOBIC002G280800	45	64	8^e-34^	SOBIC010G269000	77	82	2^e-78^
	*Triticum aestivum*	Annual	TAE32871G001	50	62	7^e-39^	TAE36408G001	73	80	9^e-75^
	*Aegilops tauschii*	Annual	XP020151150	50	78	3^e-29^	XP020177215	74	82	6^e-72^
	*Brachypodium distachyon*	Annual	BRADI4G36587	52	62	4^e-35^	BRADI1G29920	75	81	1^e-68^
	*B. stacei*	Annual	BRAST05G197700	45	47	3.7^e-18^	BRAST07G238100	77	75	5.2^e-51^
	*Setaria italica*	Annual	SEITA2G291300	49	57	1^e-43^	SEITA4G282900	81	86	6^e-84^
	*S. viridis*	Annual	SEVIR2G302300.1	43	54	7.9^e-25^	SEVIR4G295100	78	84	1.1^e-59^
	*Panicum hallii*	Perennial	PAHALB03671.1	45	59	5.4^e-20^	PAHALD00176	77	81	1.8^e-58^
	*P. virgatum*	Perennial	PAVIRJ04490.1	42	59	5.4^e-21^	PAVIRJ04759CONTIG 06,819	78	81	2.3^e-59^
	*Oropetium thomaeum*	Perennial	OROPETIUM_ 20150105_06520	66	42	5^e-17^	OROPETIUM_ 20150105_16409	77	84	3^e-82^
	*Zoysia japonica*	Perennial	ZJNSC00122.1. G00160.1	50	58	2^e-27^	ZJNSC00008.1. G00500.1	77	81	1^e-76^
	*Dicanthelium oligosanthes*	Perennial	OEL23045	43	94	1^e-29^	OEL37294	76	79	6^e-64^
	*Sasa veitchii*	Perennial	BAS04368	52	98	2^e-44^	NHF	–	–	–
	*Phyllostachys heterocycla*	Perennial	PH01000511G0500	50	59	6^e-36^	PH01001986G0070	79	82	3^e-68^
Non-Poaceae	*Zostera marina*	Perennial	ZOSMA70G00700	60	43	1^e-12^	NHF	–	–	–
	*Spirodela polyrhiza*	Perennial	SPIPO3G0017700	55	41	5^e-12^	NHF	–	–	–
	*Musa acuminata*	Annual	MAC12G0975	59	42	1^e-17^	NHF	–	–	–
	*Ananas comosus*	Perennial	ACO009346	59	42	8.8^e-10^	NHF	–	–	–
	*Phalenopsis equestris*	Perennial	PEQU26966	54	41	7^e-15^	NHF	–	–	–
	*Dendrobium catenatum*	Perennial	XP020692523.1	55	43	7^e-16^	NHF	–	–	–
	*Elaeis guineensis*	Perennial	EGU0206G0533	47	42	5^e-19^	NHF	–	–	–
	*Phoenix dactylifera*	Perennial	XP008780307	50	51	2^e-17^	NHF	–	–	–

The BLASTP analyses were performed using *O. sativa* amino acid sequences as queries. The criteria used for BLAST analyses and subsequent selection of homologs were: identities (≥ 50%), E values and coverage of the query sequences against the obtained hit sequences (≥ 60%). When multiple BLAST hits were obtained, only the top hit sequences were considered for further analyses.

*NHF* no hit found.

### Phylogenetic relationship of *BtFD1* and *BtFD2* genes with homologs obtained from other Poaceae and non-Poaceae members

The phylogenetic analysis of *BtFD1* and *BtFD2* genes with homologs obtained from Poaceae and non-Poaceae members identified three major clusters. The cluster 1 was comprised of *FD1* homologs obtained from all the Poaceae plants along with three bamboos (*B. tulda, S. veitchii, P. heterocycla*). The cluster 2 was comprised of *FD1* homologs obtained from all the non-Poaceae members, while the cluster 3 was comprised of all *FD2* homologs (Fig. 1). Cluster 1 specific for Poaceae *FD1*s was subdivided into two major sub-clusters. The sub-cluster 1 hosted *FD1* sequences obtained from annual (*Z. mays, S. bicolor, S. italica, S. viridis, O. sativa, O. brachyantha*) and perennial (*Z. japonica, D. oligosanthes, P. hallii, P. virgatum*) plants, whereas sub-cluster 2 hosted only annual plants such as *H. vulgare, T. aestivum, A. tauschii, B. distachyon, B. stacei*. The *B. tulda FD1* was placed in sub-cluster 1 along with two other bamboos *P. heterocycla* and *S. veitchii FD1* (Fig. 1). Similarly, the *FD2* specific cluster 3 was also subdivided into two sub-clusters. Here, *B. tulda FD2* was clustered with *P. heterocycla* along with other annuals and perennial plants (Fig. 1).

### Expression analyses of *BtFD1* and *BtFD2* genes in different tissues, diurnal conditions and seasons

**Figure 2:**
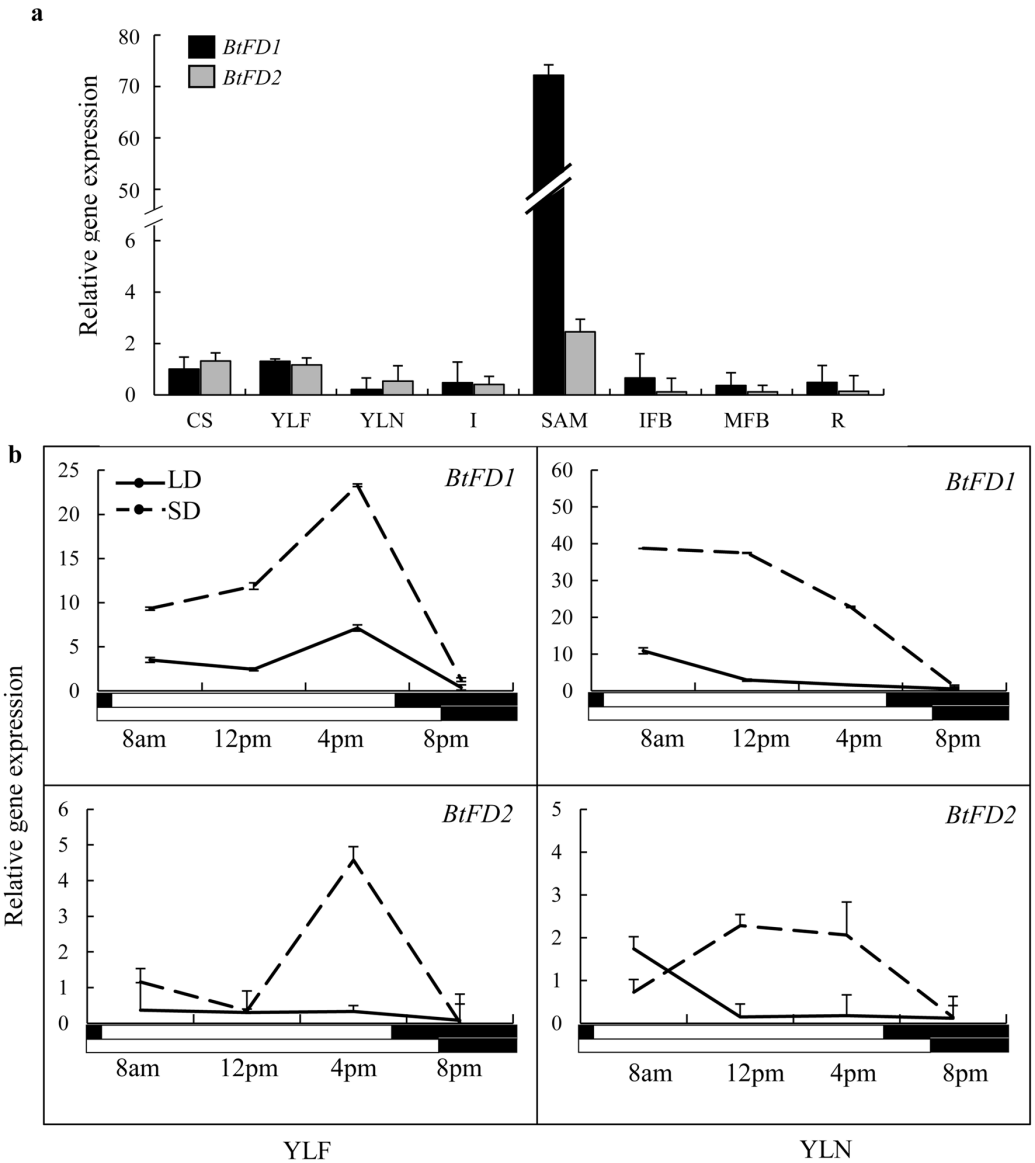
Comparison of tissue specific and diurnal expression pattern of *BtFD1* and *BtFD2* genes. (**a**) Tissue specific expressions of *BtFD1* and *BtFD2* in nine different tissue stages of *B. tulda*. Each bar represents mean of three biological replicates ± SE. (**b**) Diurnal expressions of *BtFD1* and *BtFD2* in YLF and YLN in SD and LD. Each data point represents mean of three biological replicates ± SE. Transcript expression of *eIF4α* was used to normalize expression data. The relative fold change was calculated by 2^−ΔΔCT^ method using the expression data in rhizome as calibrator and is plotted using Y axis. *CS* culm sheath, *YLF* young leaf from flowering culm, *YLN* young leaf from non-flowering culm, *I* inter node, *SA* shoot apex, *IFB* immature floral bud, *MFB* mature floral bud, *R* root.

Transcriptional expression patterns of *BtFD1* and *BtFD2* genes were investigated in diverse vegetative as well as reproductive tissues, diurnal conditions and seasons to understand the functions of these genes in bamboo vegetative as well as reproductive development. Among nine different tissues studied, expression of *BtFD1* was highest in shoot apex, followed by YLF and culm-sheath in comparison to rhizome. In contrary, the expression level of *BtFD2* was consistently very low in majority tissues studied (Fig. 2a). When diurnal expression patterns were analysed, expression level of *BtFD1* in YLF was highest in the afternoon (4 pm), which was not the case for YLN. However, the expression level of *BtFD2* was consistently very low except in a single time point i.e. afternoon (4 pm) in YLF (Fig. 2b).

**Figure 3:**
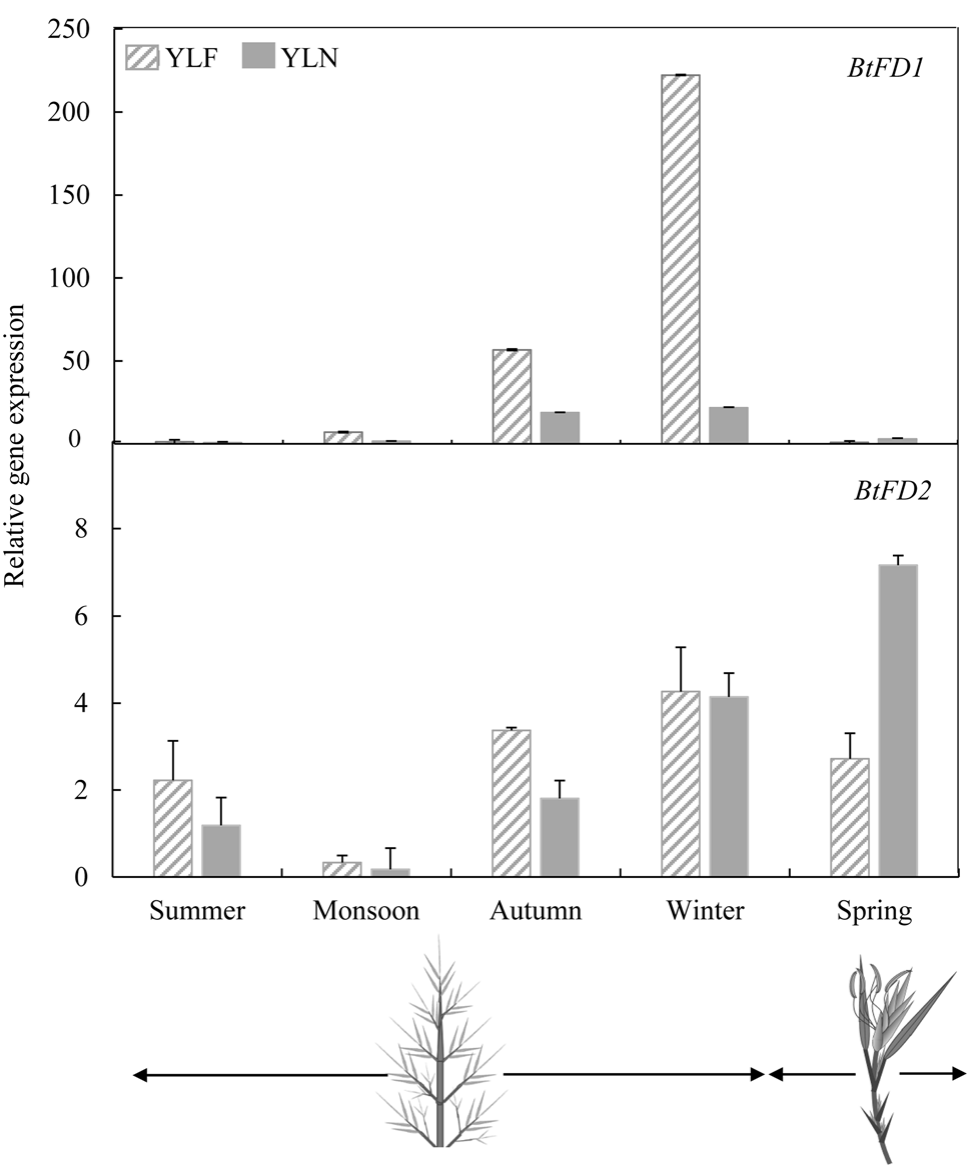
Expression analyses of *BtFD1* and *BtFD2* genes in YLF and YLN of *B. tulda* in five different seasons. Each bar represents mean expression of three biological replicates ± SE. The *eIF4α* was used to normalize expression data of the targeted flowering genes. The relative fold change was calculated by 2^−ΔΔCT^ method using the expression level observed in rhizome as the calibrator. *YLF* young leaf from flowering culm, *YLN* young leaf from non-flowering culm.

Close observation of *B. tulda* flowering habit from 2015 to 2018 revealed that sporadic flowering events usually recurred in spring every year. Therefore, to get further insight into the functions of *BtFD1* and *BtFD2* genes, their expression in young leaves were studied at three time points before onset of flowering, i.e., summer (April-June), monsoon (July–August), autumn (September–October), during onset of flowering i.e., winter (November-January) and after i.e., spring (February–March, Fig. 3). The expression level of *BtFD1* was notably higher in winter compared to other seasons (Fig. 3). In contrary, no such pattern was found for *BtFD2* expression. It was also barely detectable and quite comparable in YLF and YLN in all the seasons except a little increase in YLN in spring.

### In silico and EMSA analyses to study interaction between bZIP domains of BtFD proteins and ACGT motif

The bZIP domain of FD proteins needs to interact with the conserved CRE binding element (*ACGT*) present in the promoter of *AP1* in order to perform DNA binding activity, Therefore, the overall potential of bZIP domains present in BtFD1/BtFD2 to bind to the ACGT motif was analysed. Comparison of the bZIP domains of BtFD1, BtFD2 and their homologous sequences revealed a striking difference, *i.e.* Ala146 of BtFD1 was replaced by Leu100 in BtFD2 (Supplementary Fig. S3). In order to assess the impact of such an amino acid change, a two-pronged approach was adopted—(1) in silico prediction of overall DNA binding ability of BtFD1 and BtFD2, and (2) validation of the in silico prediction using EMSA analyses.

**Figure 4:**
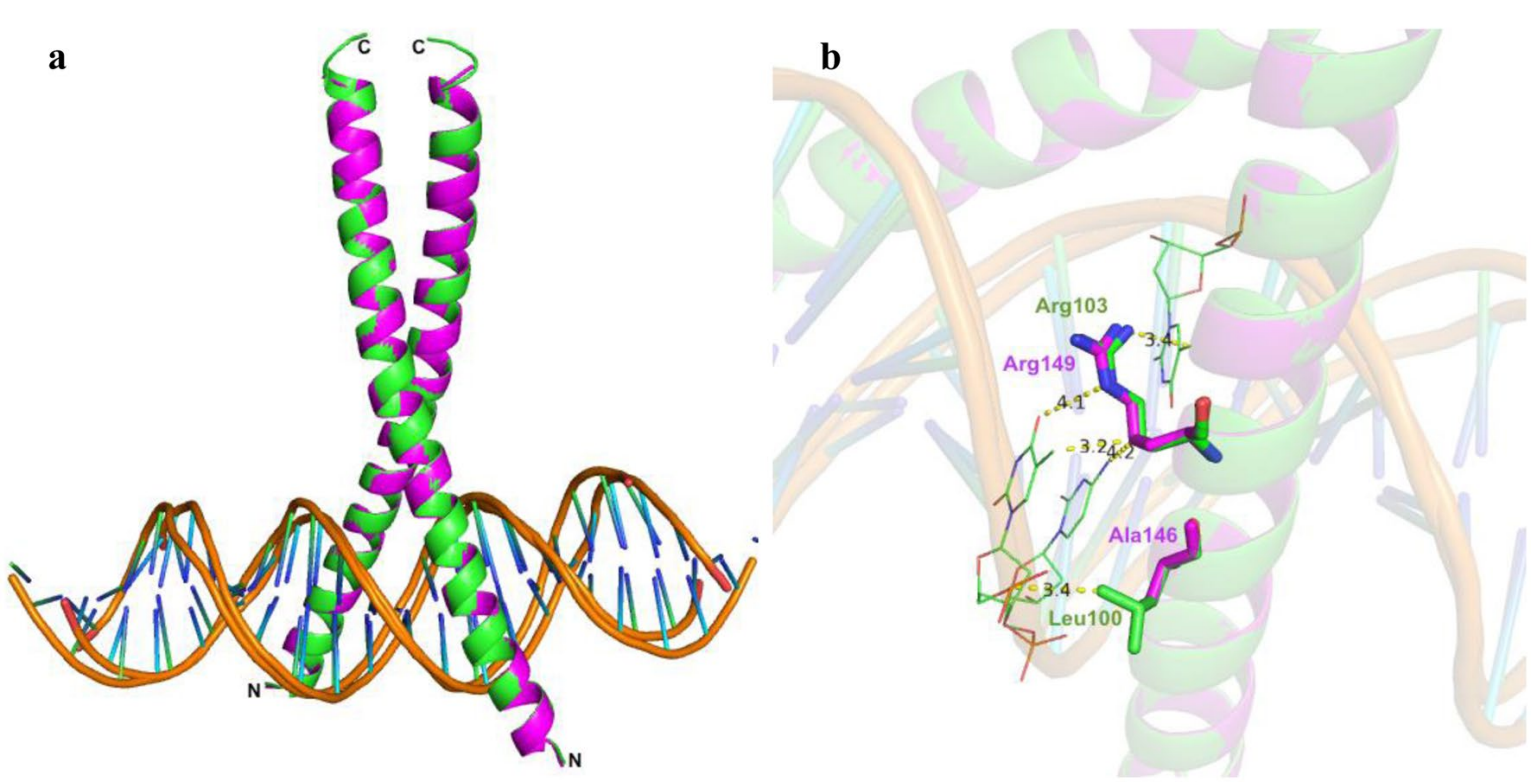
In silico interactions of bZIP domains of BtFD1 and BtFD2 with ACGT motif. (**a**) Superimposed structures of bZIP domains of BtFD1 (magenta) and BtFD2 (green) interacting with ACGT motif in a similar manner. (**b**) Arg149/Arg103 of BtFD1/BtFD2 interact with cognate DNA sequence containing ACGT motif and Leu100 of BtFD2 making additional contact with DNA (dT residue).

**Figure 5:**
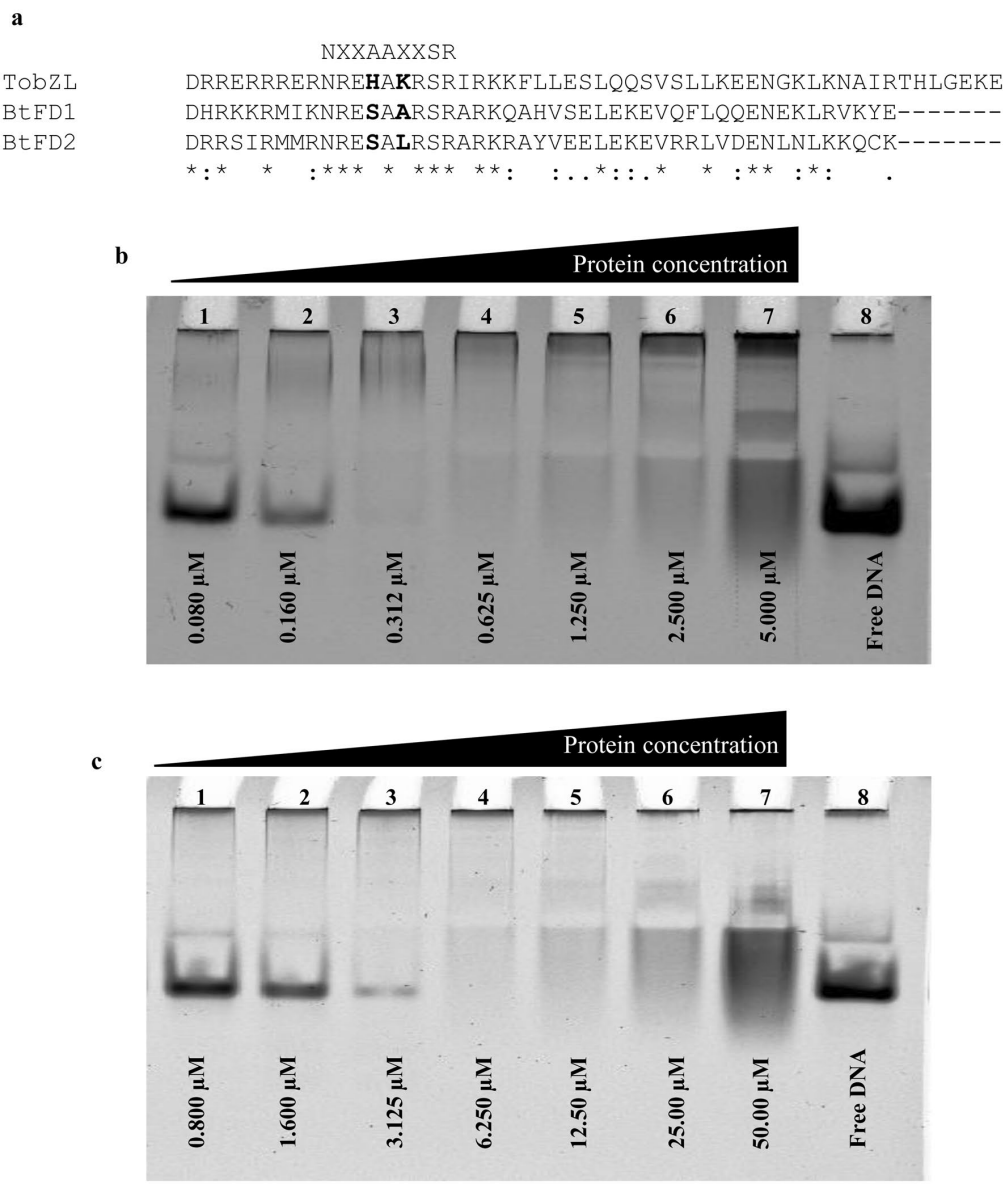
EMSA study to compare the DNA binding efficiency of bZIP domains of BtFD1 and BtFD2 proteins. (**a**) Sequence alignment of bZIP domains of TobZL (experimental template) vs. BtFD1 and BtFD2 reveals changes at two amino acids residues (His to Ser and Lys to Ala/Leu). (**b**) EMSA studies of BtFD1 and (**c**) BtFD2 mimics show overall ability to bind with CRE DNA. Lanes 1–7 in both the gels contain twofold serially diluted proteins (0.080 to 5.000 μM for BtFD1 and 0.800 to 50.00 μM for BtFD2 bZIP mimics), lane 8 contains free DNA.

Docked structures of both *BtFD1* and *BtFD2* bZIP models predicted positive interactions with CRE DNA containing ACGT motif. Superimposed docked structures also revealed that the interactions of both BtFD1 and BtFD2 could take place in a similar manner (Fig. 4a). Several amino acid residues located at the basic region, spanning from His133 to Gln153 in BtFD1 and Arg87 to Arg107 in BtFD2 were found to interact with the CRE consensus sequence. In silico docking analysis suggested that Arg142, Ser144, Arg147, Ser148 and Arg149 of BtFD1 and Arg96, Leu100, Arg101, Ser102 and Arg103 of BtFD2 were particularly found to be directly interacting with TGACGTCA consensus CRE DNA. Additionally, in silico analysis predicted direct contact for Leu100 in BTFD2 with a conserved dT residue of ACGT motif, whereas the corresponding Ala146 in BtFD1 had no interfering interactions with DNA (Fig. 4b). Even though BtFD2 gained additional interaction in this way, this non-polar-polar interaction was unfavourable in nature and therefore, could interfere with its DNA binding specificity. Ala146 on the other hand, though also non-polar, might be advantageous in this position because of its smaller size. To validate this result further, EMSA studies were conducted using mimics of BtFD1 and BtFD2 bZIPs, which only differed by a single amino acid (Ala146 of BtFD1 *vs.* Leu100 of BtFD2, Fig. 5a). Varying concentrations of bZIP mimics of BtFD1 and BtFD2 proteins were used for EMSA analysis, which showed that reappearance of free DNA begins after 0.30 µM in case of BtFD1 and 3.12 µM in case of BtFD2 (Fig. 5b,c). Free DNA is observed at 0.28 µM in case of BtFD1, which is 2.81 µM for BtFD2 (Supplementary Figs. S5a, S5b). Therefore, the concentration ranges of ‘binding to no-binding’ for bZIP mimic of BtFD1 was 0.31–0.28 µM, whereas it was 3.12–2.81 µM for BtFD2. Taken together, the finding clearly demonstrated a tenfold enhanced DNA binding specificity for BtFD1 bZIP mimic compared to its BtFD2 analogue. This means the required threshold value for BtFD2-CRE DNA interaction is much higher than that of BtFD1 (Fig. 5b,c). This further consolidated the consequence of the single amino acid substitution (Supplementary Figs. S5a–d).

### Constitutive expression of *BtFD1* and *BtFD2* genes in *Arabidopsis*

**Figure 6:**
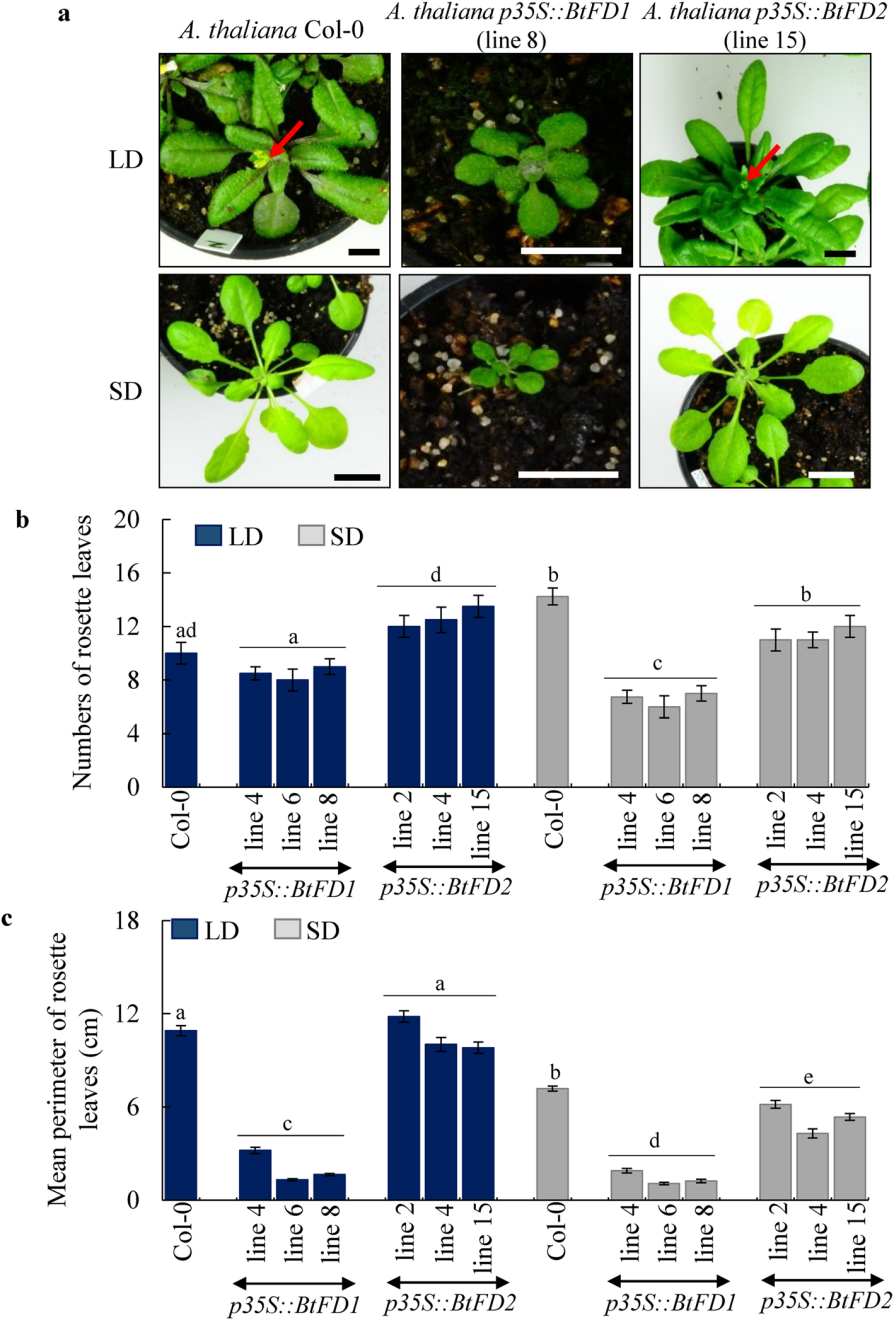
Phenotypic comparisons of wild type (WT) and transgenic *p35S::BtFD1* and *p35S::BtFD2 A. thaliana* plants*.* (**a**) Plants were grown in LD (16-h light and 8-h dark) and SD (10-h light and 14-h dark) for four weeks. Arrow indicates emerged inflorescence axis. The scale bar represents 1 cm. (**b**) Comparisons of rosette leaf numbers of four-week-old transgenic plants in (**c**) Comparisons of perimeters of rosette leaves of transgenic *vs*. WT plants. Each bar represents mean perimeter of eight individual mature leaves ± SE. comparison to WT in SD and LD. Each bar represents mean leaf numbers obtained from four individual plants ± SE. One-way ANOVA analyses were performed to test statistical significance at p.adj ≤ 0.0001. *LD* long day, *SD* short day.

In order to study roles of *BtFD1* and *BtFD2* genes on the vegetative as well as reproductive development of plants, these homologs were constitutively expressed in *Arabidopsis* (Columbia) plants. The phenotypes of transgenic *p35S::BtFD1 Arabidopsis* plants revealed drastic suppression of vegetative and floral growth in short day (SD) and long day (LD) conditions (Fig. 6a). Leaf numbers observed in three independent *p35S::BtFD1* transgenic lines after four weeks of growth were 8 to 9 in LD and 6 to 7 in SD, which were 10 and 14 in wild-type plants, respectively (Fig. 6b). The reduction in leaf number in *p35S::BtFD1* plants in comparison to WT was statistically significant in SD (p.adj = 0.000), but not in LD (p.adj = 0.193), when one-way ANOVA was conducted. In contrary, change in leaf numbers of *p35S::BtFD2* transgenic plants in comparison to WT were statistically insignificant in SD (p.adj = 0.007) as well as LD (p.adj = 0.040). Apart from the numbers, size of leaves were also significantly reduced in *p35S::BtFD1* plants in SD (p.adj = 0.000) and LD (p.adj = 0.000) compared to WT (Fig. 6c). In contrary, the difference in leaf size between *p35S::BtFD2* and WT plants were statistically significant in SD (p.adj = 0.000), but not in LD (p.adj = 0.725) (Fig. 6c). In order to simultaneously consider the effects of genetic background (WT, *p35S::BtFD1*, *p35S::BtFD2*) as well as duration of light (SD, LD), two-way ANOVA was also conducted. The genetic background had significant effect on leaf numbers (p = 0.000), whereas the light duration did not (p = 0.356). Number of leaves were significantly reduced in *p35S::BtFD1* plants compared to the WT (p.adj = 0.000). In contrary, no significant difference was obtained for leaf numbers of *p35S::BtFD2* plants compared to WT (p.adj = 0.952). However, both the genetic background (p = 0.000) as well as the light duration have significant effects (p = 0.000) on leaf perimeter. Also, change in perimeter was significant in both the cases for *p35S::BtFD1* plants compared to the WT (p.adj = 0.000), which was not the case for the *p35S::BtFD2* plants compared to the WT (p.adj = 0.022).

**Figure 7:**
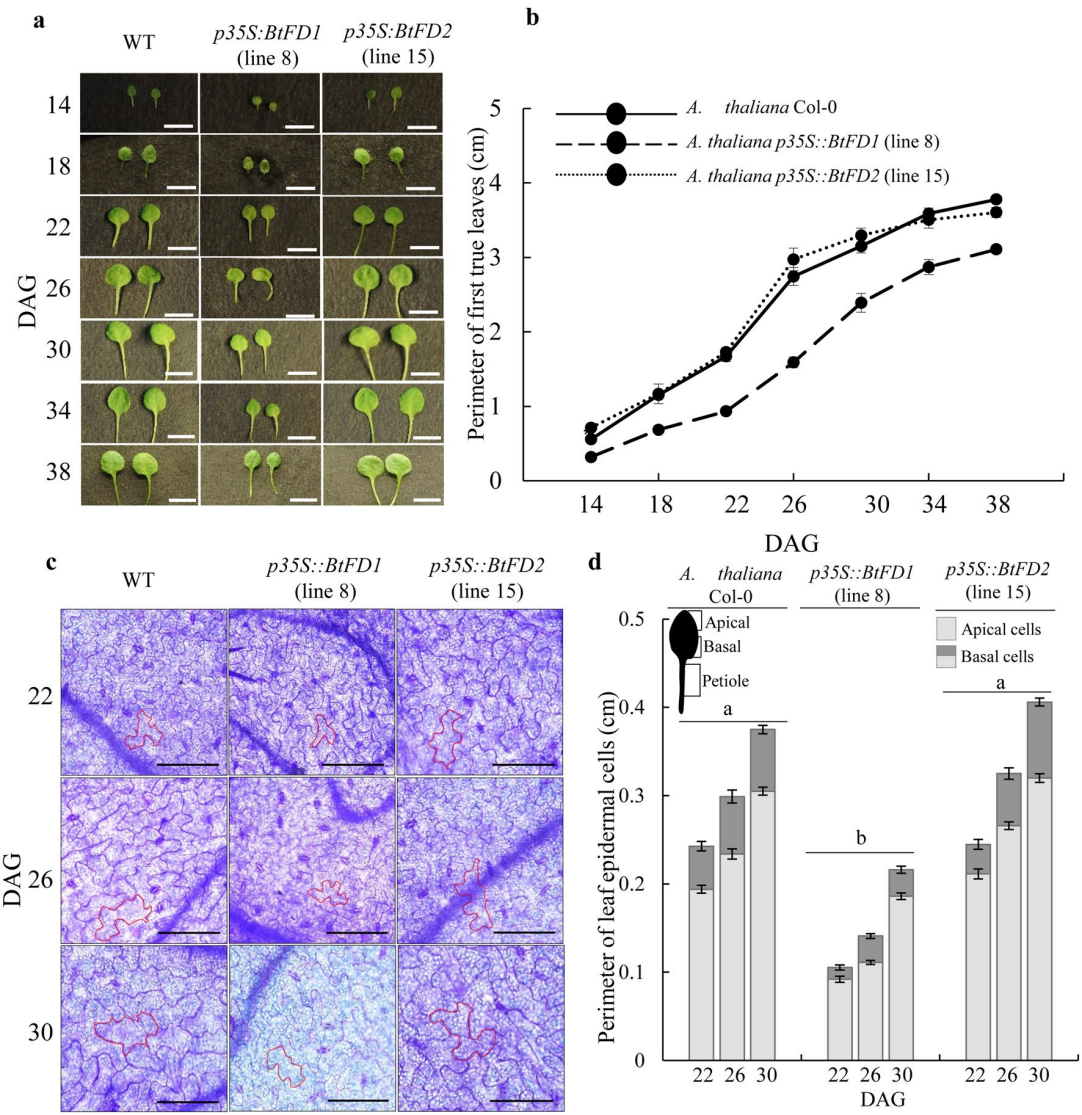
Comparison of leaf sizes of wild type *A. thaliana* (WT) with that of transgenic *p35S::BtFD1* and *p35S::BtFD2* plants. (**a**) Representative first true leaves of WT, *p35S::BtFD1* and *p35S::BtFD2* plants in 14, 18, 22, 26, 30, 34, 38 days after germination (DAG). (**b**) Perimeter of first true leaves measured in every four DAG in SD (10-h light and 14-h dark). Each data represents mean of ten individual data ± SE. The scale bar represents 5 mm. (**c**) Leaf epidermal cells obtained from the first true leaves of 22, 26 and 30 DAGs in SD. In each picture, the cell wall of a representative cell is marked by red outline. The scale bar represents 0.1 mm. (**d**) Perimeter of leaf epidermal cells measured in 22, 26 and 30 DAGs in. SD. Each data represents mean of thirty (fifteen apical and fifteen basal) individual cells ± SE. Mixed three-way ANOVA were performed to test statistical significance at p.adj ≤ 0.0001.

In order to obtain kinetic differences in leaf growth, perimeter of first true leaves were measured in four-day intervals in SD (Fig. 7a). Consistently, the leaf perimeter of *p35S::BtFD1* trasgenic plants were significantly lower than WT and *p35S::BtFD2* (Fig. 7b). Further, histological observation on leaf epidermal cells of first true leaves of these plants revealed that the perimeter were significantly lower (0.234 ± 0.005 cm to 0.299 ± 0.007 cm) in *p35S::BtFD1* (p.adj = 0.000) plants compared to WT, but not in case of *p35S::BtFD2* (p.adj = 0.066, Fig. 7c,d). Like vegetative growth, the flowering time was extremely delayed in *p35S::BtFD1 Arabidopsis* plants compared to *p35S::BtFD2* and WT (Supplementary Figs. S4a, S4b). This was apparent by the significant increase of leaf number in *p35S::BtFD1* plants compared to WT (p.adj = 0.000), but not in case of *p35S::BtFD2* (p.adj = 0.558). Additionally, the length of the flowering stalk and the numbers of flowers/plant were strongly reduced in *p35S::BtFD1* transgenic plants, while no obvious difference was noticed for *p35S::BtFD2* and WT plants in LD (Supplementary Fig. S4a). In order to promote flowering, *FD* binds to *AP1* to induce it at the transcriptional level. Therefore, the expression of *AtAP1* was measured in the wild type, *p35S::BtFD1*, and *p35S::BtFD2 Arabidopsis* plants*.* Indeed, the expression of the *AtAP1* gene in the four-week-old leaves of *p35S::BtFD1 Arabidopsis* plants grown under LD was tenfold higher compared to WT, which was only twofold in case of *p35S::BtFD2* plants (Supplementary Fig. S4c).

## Discussion

### Bamboo *FD* genes are similar to other Poaceae *FD* homologs in terms of sequence similarity and phylogenetic relationships

*FD* is a bZIP family protein and plays important roles in controlling the timing of reproductive phase transition in angiosperms^29,33,45^. In addition, its role in vegetative development has also been observed^29^. In this study two bamboo *FD* genes (*BtFD1* and *BtFD2*) were identified and their sequences were characterized to study phylogenetic relationships of these genes to other homologous monocot genes*.* Characterization of the amino acid sequences inferred that like other Poaceae *FD1*s, bamboo *BtFD1* possessed motifs *1*, *LSL*, *bZIP* and *SAP*, but not motif *4*, which is usually characteristic of non-Poaceae *FD1*s^29^ (Supplementary Fig. S2). Among these motifs, *bZIP* and *SAP* were found absolutely necessary for the interaction with *AP1*^36^ and *14–3-3*, respectively. However, the functional significance of other motifs in flowering needs further investigation^33^. In contrary, the *LSL* motif is absent in all *FD2* homologs including bamboo that have been identified so far suggesting their less likely involvement in flowering (Supplementary Fig. S2).

Phylogenetic analyses of the *FD* homologs obtained from monocotyledonous plants revealed the presence of three major clusters. Cluster 1 and 2 were comprised of *FD1* homologs of Poaceae and non-Poaceae members respectively, while all *FD2*s from the Poaceae species were placed in cluster 3 (Fig. 1). All the bamboo *FD1* and *FD2* homologs (*P. heterocycla, B. tulda* and *S. veteichii*) were found in the Poaceae specific clades of *FD1* and *FD2* respectively (Fig. 1). It had previously been found that the *FD* gene clade could be broadly classified into four subgroups, which were Poaceae specific *FD1*, Poaceae specific *FD2*, Poaceae specific *FD3* and *FD*s obtained from eudicots as well as non-Poaceae members of monocots^29^.

### Bamboo *FD1 *and *FD2* genes are divergent in expression patterns with respect to tissues, diurnal conditions and seasons

The detailed expression analyses of *BtFD1* and *BtFD2* genes in diverse tissues, diurnal conditions and seasons may provide clues about their possible functionality. It is well established that in SAM, *FT* interacts with *FD* to form FAC and consequently floral meristem identity genes are activated to induce flowering^45^. Therefore, *FD* expression has primarily been observed in SAM tissues of *Arabidopsis,* rice, *P. sativum*, *P. tremula x P. alba* and *P. aphrodite* plants^29,30,33,40,42,46^. In addition, expression of *FD1* was also detected in leaves of *O. sativa*^29^, *T. aestivum*^36^ and *A. chinensis*^41^ plants. In bamboo, the expression level of *BtFD1* gene was highest in shoot apex. However, the expression of *BtFD2* was very low in all the tissues. This is unlike rice, where *FD2* was primarily expressed in leaves^29^. Like many other flowering genes, *FD* also was found diurnally regulated in rice^34^ and *P. tremula x P. alba*^42^. In bamboo, expression of *BtFD1* in YLF was highest in the afternoon (4 pm), but in YLN it was in the morning (8am). In poplar, similar diurnal regulation of *FD* was observed in SD, where it attained its maximum expression at mid night, whereas no such pattern was observed in LD^42^. In contrary, the diurnal expression of *BtFD2* remained consistently low throughout the day. Expression analyses across seasons also point towards a role of *BtFD1*in flower induction. Transcript accumulation of *BtFD1* in the floral inductive tissue YLF began in autumn and reached the maximum level in winter, *i.e.* just before sprouting (Fig. 3). This observation was comparable to perennial dicots poplar and kiwifruit, where *FD* was transiently expressed just before flowering every year^41,42^. In contrary, expression of *BtFD2* was almost negligible throughout the year. Taken together, the analysed expression data suggest that *BtFD1* may perform important roles in flower and vegetative development of bamboo, whereas the function of *BtFD2* is yet to be discovered.

### A single amino acid change resulting into differential binding efficiency between bZIP domains of *BtFD1/BtFD2* and CRE DNA

Sequence analyses and in silico characterization of the two BtFD proteins confirmed that they belong to bZIP transcription factor family. Among several different subfamilies of bZIPs, BtFDs were found to be homologous to CREB. Co-crystal structure of CRE DNA—CREB bZIP of *Mus musculus*^35^ (PDB ID IDH3) was chosen for homology modelling purpose. Generally, the CREB family bZIP members are capable to interact with A box (TACGTA), G box (CACGTG) or C box (GACGTC) elements present in the promoter region of their target genes causing transcriptional upregulation^47,48^. In plants, the FD1 members of CREB family are involved in the establishment of floral meristem identity^30,36,39^. Overall, the bZIP regions of BtFD1 and BtFD2 proteins differ in five different amino acid positions. Particularly one position (Ala146 is BtFD1 *vs*. Leu100 in BtFD2) at the crucial DNA binding site (NXXAAXXSR) was interesting. Therefore, it was asked whether any of these amino acid changes, in particular this single amino acid substitution, could have any impact on their DNA binding activity. Our in silico analyses revealed that the bZIP domains identified in BtFD1 and BtFD2 were capable to dimerise and form a bZIP structure. They also demonstrated specific interaction with the TGACGTCA sequence. In particular, Asn141, Arg149 of BtFD1 and Asn95, Arg103 of BtFD2 can directly interact with cognate DNA substrate. Similar interaction has also been found in maize^39^ and wheat^36^. EMSA analysis highlighted that the change of Ala146 in BtFD1 *vs*. Leu100 in BTFD2 resulted into ten times enhanced binding of BtFD1 than BtFD2. This may be the result of an additional, yet unfavourable contact between Leu100 of BtFD2 and dT residue of ACGT motif, apparent in the docked structure. It might be possible that Leu100 interfered with the interaction of target DNA by making a polar *vs*. non-polar interaction. In contrary, the shorter Ala146 residue, which was present in BtFD1 could not interfere and thus enabling higher DNA binding efficiency of BtFD1 (Fig. 5b,c).

### Ectopic expression of *BtFD1* severely suppressed vegetative growth and flowering in *Arabidopsis*, but *BtFD2* did not

In order to study the functions of *BtFD1* and *BtFD2* genes *in planta*, transgenic alterations of these genes needed to be carried out. Altering activities of these genes in bamboo itself were difficult due to many reasons such as long-life cycle, difficulty with in vitro regeneration and unavailability of efficient transformation methods^49,50^. Therefore, *BtFD1* and *BtFD2* genes were ectopically expressed in *Arabidopsis* plants and their phenotypes were compared.

The vegetative growth of transgenic *Arabidopsis* plants overexpressing *BtFD1* gene was severely suppressed with respect to the number and size of the rosette leaves (Fig. 6a–c). Similar phenotypes had been noticed when *AtFD* and *AtFDP* together were overexpressed in rice^45^ and also when poplar *FDL1* was overexpressed in *Populus tremula* × *tremuloides*^42,51^. The involvement of *FD* in controlling vegetative growth has been observed in a pea loss-of-function mutant, demonstrating severe branching even after flower induction^40^. A few molecular players in connection to the growth retardation due to *FD1* overexpression have been identified. For instance, in poplar*, BRANCHED1* and *2* genes, which promote shoot growth by maintaining proper auxin and cytokinin levels were downregulated^52,53^. Overexpression of *Arabidopsis FD* and *FDP* in rice resulted in the down-regulation of many cell wall growth responsive genes such as *EXTENSIN*, *EXPANSIN* and *XTH1*^45^. Similar to all these previous observations, in this study the *p35S::BtFD1 Arabidopsis* plants revealed reduced leaf and leaf epidermal cell sizes compared to *p35S::BtFD2* and wild-type plants.

The role of *FD1* in flower induction has already been established by a large body of literature and a variety of mutant phenotypes have been observed: (a) Delay in flowering was observed in the loss-of-function mutants of *Arabidopsis*^30,37,38^, pea^40^ and maize^39^, while early flowering was observed in the *FD1* overexpressing lines of rice^29,33^ and *Phalaenopsis*^46^. (b) However, exceptions to this line of observation have also been noticed^42,45,51^. When *AtFD* and *AtFDP* together were overexpressed in rice, flowering and vegetative growth has been retarded^45^. Similarly, overexpression of poplar *FDL1* in *Populus tremula* × *tremuloides* resulted into delayed flowering in SD^42,51^. Our results demonstrated that transgenic *Arabidopsis* plants overexpressing *BtFD1* exhibited a delay in flowering time and numbers of flowers/plant compared to *p35S::BtFD2* and WT plants (Supplementary Fig. S4a). However, expression of *AtAP1* was remarkably higher in *p35S::BtFD1 Arabidopsis* than *p35S::BtFD2* and WT plants. Similar observations were also reported in *AtFD* and *AtFDP* overexpressing lines of rice^45^ and *FDL* overexpressing lines of poplar^51^, which, nevertheless, led to late flowering phenotypes. Together, it can be concluded that timely expression of *BtFD1* may be critical to perform its programmed flower specific role *in planta*, which was altered in the transgenic *Arabidopsis* plants constitutively overexpressing *BtFD1* in a spatially and timely improper manner. Therefore, the apparent delay in flowering time could be an indirect effect of extensive suppression of vegetative growth, while in contrast, the flowering program is still enhanced based on the marker gene *AtAP1* induction. It is already well accepted that flowering can only be induced after plants attain sufficient vegetative growth^54^.

The evolution of gene function within the *FD* family revealed the existence of functional redundancy in *Arabidopsis*^45^. In contrary, a clear functional diversification was noticed between the two rice *FD* genes *OsFD1 vs*. *OsFD2*^29^. Our study revealed that the two bamboo *FD* genes imposed contrasting effects on shoot growth and flowering time, which may be mediated by two ways: (a) by acquiring expression divergence where *BtFD1* maintained a flower associated expression pattern whereas expression level of *BtFD2* was consistently low and (b) by adapting a single amino acid change (Ala146 *vs*. Leu 100) located in their DNA binding region which may cause a differential binding to their target protein AP1. Future studies are required to investigate the impact of residue alterations in the other four positions. Such single residue swapping was found sufficient to convert the flowering repressor *TFL1* to an activator *FT* and vice versa by altered interaction with their interactor proteins^55^. Taken together, it can be concluded that regions involved in protein–protein or DNA–protein interactions can be potential targets to study the functional evolution of closely related homologous genes. Further studies are required to uncover whether *BtFD1* is anyhow involved in long perennialism of bamboo and whereas its homolog *BtFD2* evolved any additional function or required other interacting partner to be functional.

## Materials and methods

### Collection of *Bambusa tulda* tissues for gene expression analyses

Flowering tissues were obtained from three populations of *B. tulda* located in Shyamnagar, W.B. (SHYM7, SHYM16, 22.38° N. 88.40° E) and Bandel^11^ (BNDL22, 22.93° N. 88.38° E). Recurrent incidence of sporadic flowering was noticed every year in spring from 2015 to 2018. Corresponding voucher specimen were submitted to the Botanical Survey of India (B.S.I), Shibpur (deposition nos. 56A, 56B, 57A, 57B, 58A. 58B, 59A, 59B, 59C on 05.06.2015). To perform tissue specific gene expression analysis, six vegetative tissues such as young leaf from flowering (YLF) and nonflowering culm (YLN), culm sheath (CS), root (R), internode (I), shoot apex (SA) and two flowering tissues such as immature and mature floral buds (IFB, MFB) were collected. In order to perform diurnal expression analyses, YLF and YLN were collected at four different time points of a day- morning (8 am), noon (12 pm), afternoon (4 pm) and night (8 pm) for both long day (LD, 14 h light exposure, sunrise at 4:30 am and sunset at 6:30 pm) and short-day (SD, 11 h light exposure, sunrise at 6 am and sunset at 5 pm). Tissues were also collected in five different seasons: summer (April–June, 2017), monsoon (July–August, 2017) autumn (September–October, 2017), winter (November–January, 2017) and spring (February–March, 2018). At least three, independent biological replicates were used for each tissue stage/diurnal condition/season.

### Isolation of nucleic acids and preparation of cDNA libraries

Isolation of genomic DNA was carried out from young, healthy leaves by using DNeasy Plant Mini Kit (QIAGEN, Germany). Total RNA was isolated by a combination of Trizol (INVITROGEN, USA) and RNAeasy Plant Mini Kit (QIAGEN, Germany) as per the procedure described before^43,56^. Samples were treated with DNase I enzyme (THERMO SCIENTIFIC, USA) to avoid genomic DNA contamination, if any. Quality and quantity of the samples were checked in a BioSpectrometer (EPPENDORF, Germany) and agarose-formamide gel elctrophoresis. Approximately 1 μg of total RNA was used for cDNA synthesis using verso cDNA synthesis kit (THERMO SCIENTIFIC, USA) following manufacturer’s protocol. For real time RT-qPCR analyses, 2 μl of tenfolds diluted stock solution of cDNA samples was used.

### Analysing *FD* gene and amino acid sequences obtained from various genome databases

Rice gene sequences (*OsFD1: OS09G36910* and *OsFD2: OS06G50830*) were used as queries to retrieve genomic as well as amino acid sequences of *FD1* and *FD2* genes available in various genome databases. BLASTP analyses were performed in Phytozome (https://phytozome.jgi.doe.gov/pz/portal.html), Plaza_monocot_v4 (https://bioinformatics.psb.ugent.be/plaza/versions/plaza_v4_monocots/) and NCBI (https://www.ncbi.nlm.nih.gov) databases. All BLASTP hits were obtained using the set criteria of an E-value threshold ≤ e^−10^, identity ≥ 40% and length coverage with respect to the query sequence ≥ 40%. However, when multiple hits were obtained, only the best BLASTP hit was selected for further analyses. If no homologous genes were found using the set criteria, it is mentioned as ‘no hit found’ (NHF, Table 1).

### Primer designing, PCR amplification and sequencing of *B. tulda BtFD1* and *BtFD2* genes

In order to obtain *B. tulda* genes, homologous sequences obtained from closely related monocot species were aligned and degenerate primers were designed from the conserved regions by using Primer3 program (http://bioinfo.ut.ee/primer3-0.4.0/, Supplementary Table S1). PCR amplification was conducted using high fidelity Phusion Taq DNA polymerase (THERMO SCIENTIFIC, USA). Amplified PCR products of desired molecular weight were gel purified by using GeneJET gel elution kit (THERMO SCIENTIFIC, USA) and cloned into TA vector (pGEM-T Easy Vector Systems, PROMEGA, USA) or blunt end vector (pJET PCR cloning kit, THERMO SCIENTIFIC, USA). Selection of bacterial colonies were done based on the blue-white screening and/or ampicillin sensitivity (100 µg/ml). Plasmids were isolated by GeneJET plasmid miniprep kit (THERMO SCIENTIFIC, USA). Sanger’s sequencing was undertaken and contigs were assembled by CAP3 (www. http://doua.prabi.fr/software/cap3) prior to submission to NCBI (*MF983712*, *MH142577*). The full length genomic and coding sequences (CDS) were analysed in the Gene Structure Display Server (GSDS, http://gsds.cbi.pku.edu.cn/index.php) to predict the gene models.

### Sequence data and phylogenetic analyses

The *FD* gene sequences identified from *B. tulda* were used as query and BLASTP analyses were performed in NCBI (https://www.ncbi.nlm.nih.gov) database to identify its homologous sequences in related species. Amino acid sequences of *BtFD1* and *2* genes were aligned with other homologous sequences using the Clustal Omega program (https: //www.ebi.ac.uk/Tools/msa/clustalo/). A phylogenetic tree was constructed by using the Neighbour Joining (NJ) method in MEGA7 tool^57^. Bootstrap analysis with values for 2000 replicates was conducted to estimate nodal support.

### In silico modelling and docking studies

In silico analysis was performed to predict the possibility of binding between of bZIP domains of BtFD1 or BtFD2 to the ACGT motif. Due to unavailability of BtFD1 and BtFD2 crystal structures, amino acid sequences corresponding to their bZIP domains were first subjected to homology modelling by SwissModel (https://swissmodel.expasy.org). Both of them demonstrated significant sequence homology (36% identity) with their nearest structural homologue CRE binding protein known from *Mus musculus*^35^ (PDB ID IDH3). Therefore, it was chosen as template for modelling bZIPs of BtFD1 and BtFD2. Ramachandran analysis using Molprobity option from Swissmodel revealed 98.96% residues to be in favourable region for BtFD1 and 95% for BtFD2 and both were modelled in their dimeric state. The two bZIP models were then subjected to energy minimization using Chimera (https://www.cgl.ucsf.edu/chimera/). Ramachandran analysis through Procheck (https://servicesn.mbi.ucla.edu/PROCHECK/) and post energy minimization revealed complete inclusion of residues in favourable region for both the models. The energy minimized structures were then docked with 21 bp CRE DNA sequence (obtained from 1DH3 crystal structure) using NPdock (http://genesilico.pl/NPDock).

### Site-directed mutagenesis for electrophoretic mobility shift assay (EMSA)

In order to validate the prediction of binding between bZIP domains of BtFD1 and BtFD2 with the ACGT motif, electrophoretic mobility shift assay was performed. The bZIP sequence obtained from *Thalassiosira oceanica* LOV photoreceptor (To_bZIP + LOV − TobZL) protein, was used for site directed mutagenesis to obtain bZIP mimics of BtFD1 and BtFD2 proteins. Pairwise sequence alignment between among bZIP regions of BtFD1, BtFD2 and TobZL revealed two amino acid differences at the DNA binding basic region (Fig. 5a**)**. Double mutations leading to conversion of His > Ser144 and Lys > Ala146 were introduced in TobZL to mimic *BtFD1* bZIP and His310 > Ser98 and Lys312 > Leu100 to mimic *BtFD2* bZIP. Mutations were done using standard procedures to induce site directed mutagenesis and were verified by DNA sequencing (EUROFINS GENOMICS INDIA PVT. LTD).

### Over-expression and purification of *BtFD1* and *BtFD2* bZIP mimics

The *BtFD1* and *BtFD2* bZIP mimics were cloned in pET28a expression vector, transformed in *E. coli* [BL21(DE3)C43] and grown at 37 °C. After isopropyl β-D-1-thiogalactopyranoside induction, bacterial cells were grown at 20 °C for overnight. Cells were then centrifuged and pellets were re-suspended in buffer containing 20 mM Tris (pH 8.0), 10 mM NaCl, 10% glycerol in presence of the protease inhibitor. Following sonication on ice and centrifugation, the supernatant was incubated with Ni–NTA agarose (QIAGEN, Germany) for 2 h. After washing in 10 mM imidazole containing re-suspension buffer, proteins were finally eluted with 250 mM imidazole. The eluted fractions were next pooled and excess imidazole was removed using PD10 desalting column (SIGMA). Proteins were concentrated and stored at − 20 °C in aliquots for future use.

### Electrophoretic mobility shift assay

Electrophoretic mobility shift assay was carried out to study DNA-binding activity of *BtFD1* and *BtFD2* proteins. A 24 bp DNA fragment [5′ d(TGTAGCGTCTGACGTGGTTCCCAC) 3′ and complementary sequence] containing the consensus CREB binding site, TGACGT, were synthesized (INTEGRATED DNA TECHNOLOGIES). Lyophilized DNA strands (labeled and un-labelled) were suspended in nuclease free water and 10 μM of it was annealed by rapid heating at 95 °C followed by gradual cooling in annealing buffer (12 mM Tris; pH 8.0, 30 mM NaCl). A final concentration of 0.5 μM double stranded DNA was used in the protein DNA binding assay buffer (50 mM Tris–HCl pH 8.0, 20 mM NaCl, 0.5 mM DNA, 1.25 mM MgCl_2_, 20% glycerol). Serially diluted protein was added to the solution followed by an incubation at 22 °C for 1 h. The protein DNA complex along with the control set (only DNA) was resolved in 10% polyacrylamide gels at 180 V for 35 min. The gel was then stained with SyBr Gold (THERMO SCIENTIFIC, USA) in 0.5X TBE buffer for 40 min and imaged using a gel documentation system (BIORAD, USA).

### Gene expression analyses by real time RT-qPCR

To perform real time RT-qPCR analyses, gene specific primers were designed from the coding sequences of the *BtFD1* and *BtFD2* genes using Primer3 program (http://bioinfo.ut.ee/primer3-0.4.0/, Supplementary Table S1). The real time RT-qPCR analyses were performed by using SsoAdvanced Universal SYBR Green Supermix (BIO-RAD, USA) and CFX connect real-time PCR detection system (BIO-RAD, USA). To confirm the absence of any primer dimers in the amplified products, a standard melt curve analysis was conducted. The *BteIF4α* and *AtACT2* genes were previously identified as ideal reference gene for normalizing expression data obtained from *Bambusa*^58^ and *Arabidopsis*^56^, respectively. The relative fold change in gene expression level was calculated by the 2^−ΔΔCt^ method^59^. The PCR amplification efficiency were measured for the five pairs of primers used in RT-qPCR. Two fold serial dilutions of the pooled cDNA templates were used to obtain standard curves for each primer pair. The amplification efficiency was analyzed using the formula^60^ 10^(−1/slope)^ − 1 × 100. The obtained percentage of efficiency was 95%-98%.

### Gateway cloning of *BtFD1* and *BtFD2* genes

Gateway recombination sequences were tagged to the 5′ end of the primers to PCR amplify *BtFD1* and *2* genes using Phusion Taq DNA polymerase enzyme (THERMO SCIENTIFIC, USA, Supplemental Table S1). Approximately 100 ng of gel-purified PCR fragments were recombined with 100 ng of pDONR221 donor vector using BP Clonase enzyme (INVITROGEN, USA). Reactions were transformed into *E. coli* (*DH5α*) and isolated plasmids were verified by DNA sequencing before recombination into the binary pAlligator2 vector providing The CaMV 35S promoter for expression^61^. Finally, the expression clones were mobilized to competent *Agrobacterium tumefaciens* (pGV3101/pMP90) by electroporation using a BIO-RAD Gene Pulser.

### *In planta* transformation, selection, phenotypic characterization and statistical analysis

Approximately six-week-old *A. thaliana* (Col-0) plants were transformed by the floral dipping method^62^. Transformed T_1_ seeds were selected on the basis of green fluorescence of the GFP reporter gene^61^. The number and perimeter of the rosette leaves were measured from three independent T_3_ plants having single insertions in order to perform phenotypic comparisons with wild-type *A. thaliana* Col-0 plants grown in both long day (LD, 16-h light and 8-h dark) and short day (SD, 10-h light and 14-h dark) conditions for four weeks. One-way ANOVA was carried out in R software (version 3.4.4) to find the degree of significance with respect to the difference in leaf numbers and sizes among *p35S::BtFD1*, *p35S::BtFD2* and WT plants. For the analyses of leaf number, twelve replicates were considered for each of *p35S::BtFD1* and *p35S::BtFD2* transgenic plants (3 transgenic lines and 4 individual plants), whereas for WT it was 4. For the analyses of leaf perimeter, 24 replicates were considered for each of the *p35S::BtFD1* and *p35S::BtFD2* transgenic plants (3 transgenic lines and 8 individual leaves), whereas for WT it was 8. Since comparisons among three genetic backgrounds of plants (WT, *p35S::BtFD1* and *p35S::BtFD2*) were performed in pairs (3 pairs), adjusted p-values (Tukey’s HSD) were considered and expressed as p.adj. In case of two-way ANOVA, one factor was considered as the genetic background (WT, *p35S::BtFD1* and *p35S::BtFD2*), whereas the other factor was the duration of light (LD vs. SD). Here also, adjusted p-values (Tukey’s HSD) were used for conducting pairwise comparisons among three genetic backgrounds of plants (WT, *p35S::BtFD1* and *p35S::BtFD2*). In order to study if there is any significant change in flowering time among WT, *p35S::BtFD1* and *p35S::BtFD2* plants, total number of rosette leaves were counted during the time of flowering from 6 independent plants/genetic background and one-way ANOVA was carried out to test significance in difference. The adjusted p values were obtained via Tukey’s HSD.

In order to obtain kinetic pattern of the differences in leaf growth, the perimeter of the first true leaves were measured in four day intervals in SD by using photographs and ImageJ software^63^. In addition, histological observation was performed on leaf epidermal cells since it had previously been observed that a positive correlation exists between expansion of leaf lamina and size of epidermal cells^64^. It was observed in the light microscope using NIS elements software and DS-Qi2 NIKON camera and the perimeter of epidermal cells were obtained from the apical and basal parts of the first true leaves of 22-, 26-, and 30-day-old plants grown in SD. Ten epidermal cells obtained from leaves of three independent plants of WT, *p35S::BtFD1* and *p35S::BtFD2* were subjected to mixed three-way ANOVA to test significance in difference of epidermal cell sizes. The adjusted p values were obtained via Bonferroni correction. In order to observe epidermal cells in the light microscope, first true leaves were preserved in 10% formaldehyde: 50% ethanol: 5% acetic acid solution. Leaves were dipped in absolute ethanol and boiled for 30–45 s to remove chlorophylls and were subsequently stained with 0.01% toluidine blue.

## Supplementary Information

Supplementary Information 1.

Supplementary Information 2.

Supplementary Information 3.

Supplementary Information 4.

Supplementary Information 5.

Supplementary Information 6.
