# Supplementary figures and images for: Identification and functional characterization of two bamboo FD gene homologs having contrasting effects on shoot growth and flowering
Source: Sci Rep. 2021 Apr 12;11:7849. doi: 10.1038/s41598-021-87491-6 (PMC8041875; doi:10.1038/s41598-021-87491-6)

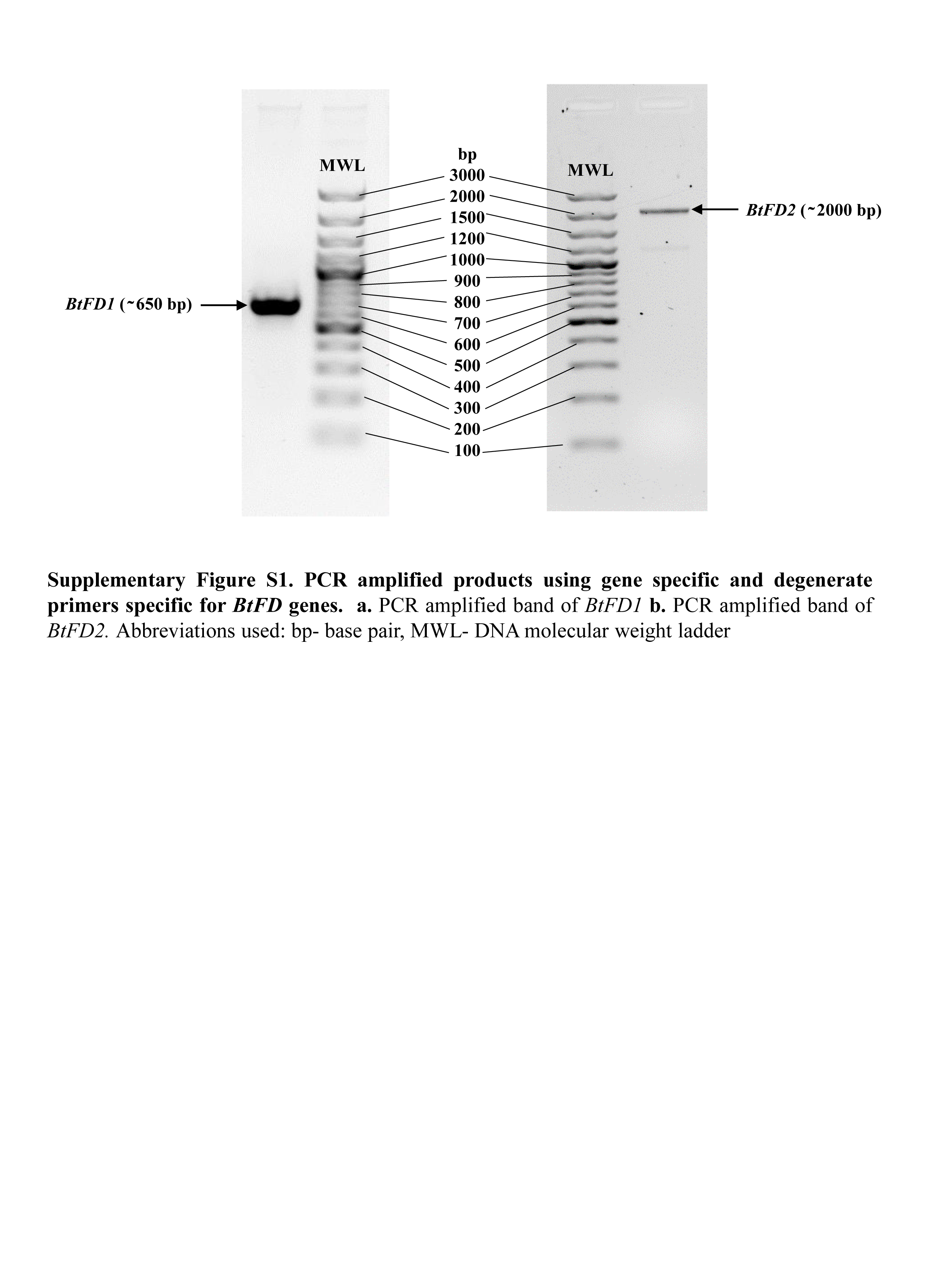

Supplement: Supplementary file 1 — Supplementary Information 1. [file 41598_2021_87491_MOESM1_ESM.tif]

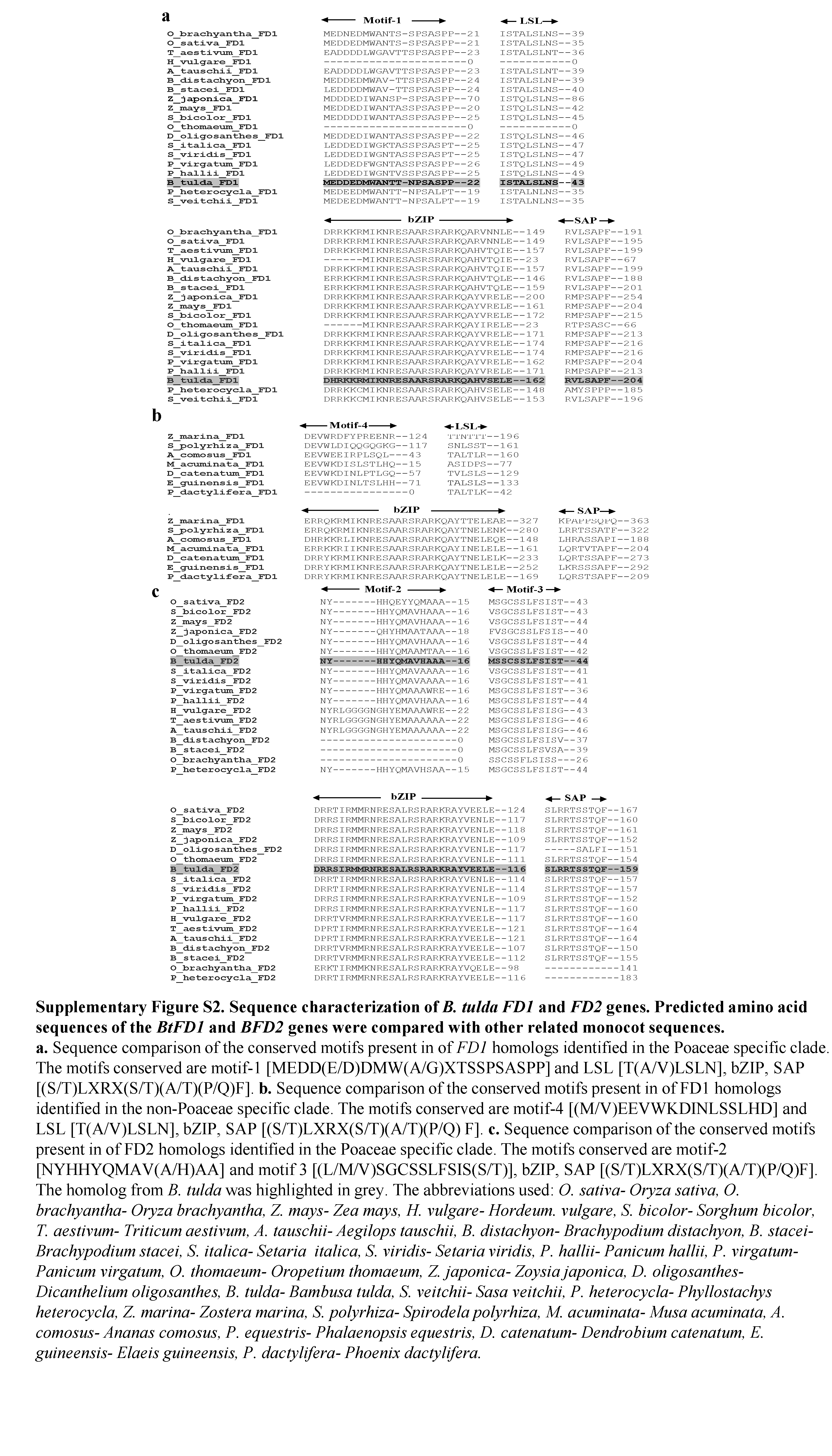

Supplement: Supplementary file 2 — Supplementary Information 2. [file 41598_2021_87491_MOESM2_ESM.tif]

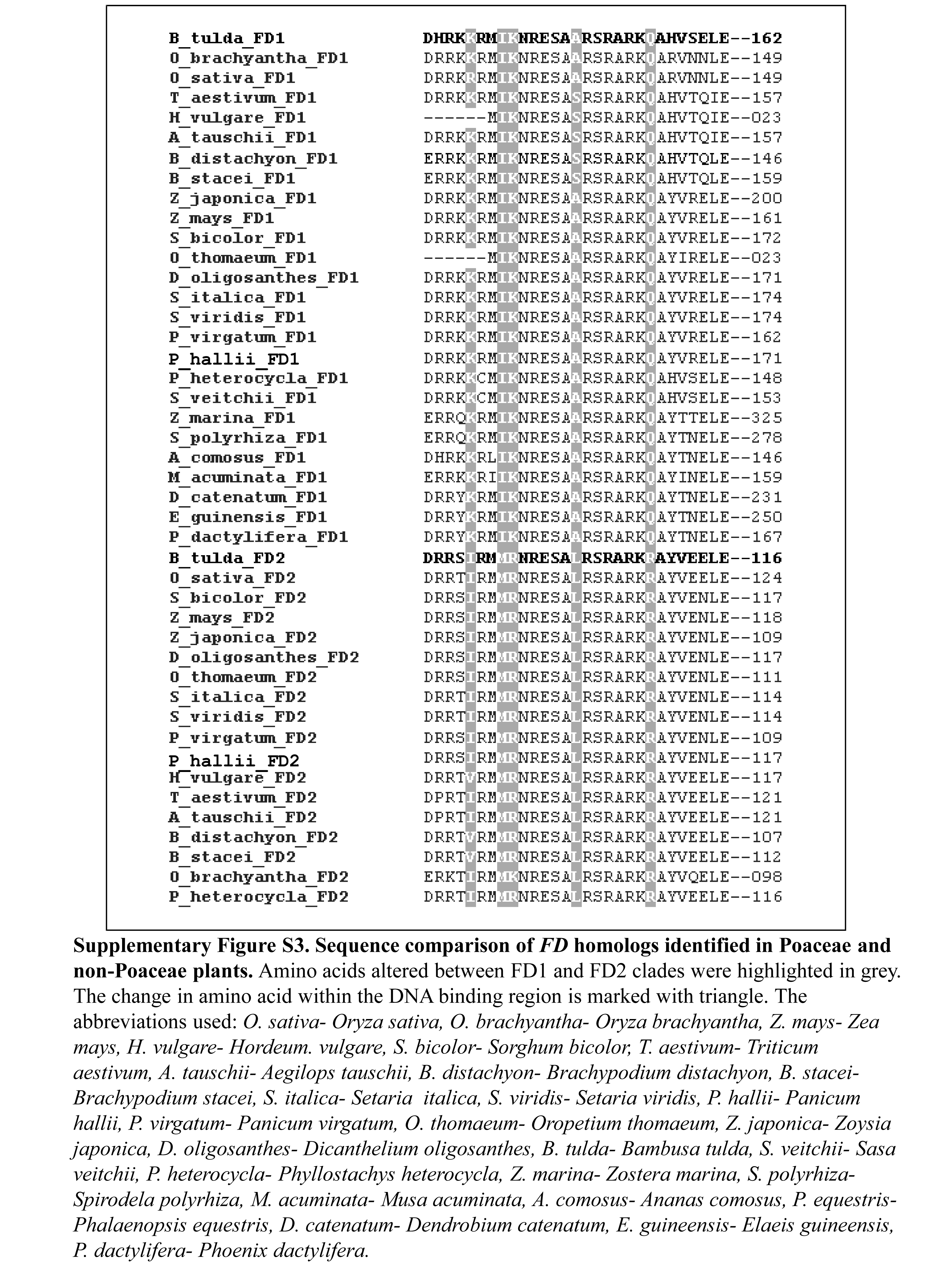

Supplement: Supplementary file 3 — Supplementary Information 3. [file 41598_2021_87491_MOESM3_ESM.tif]

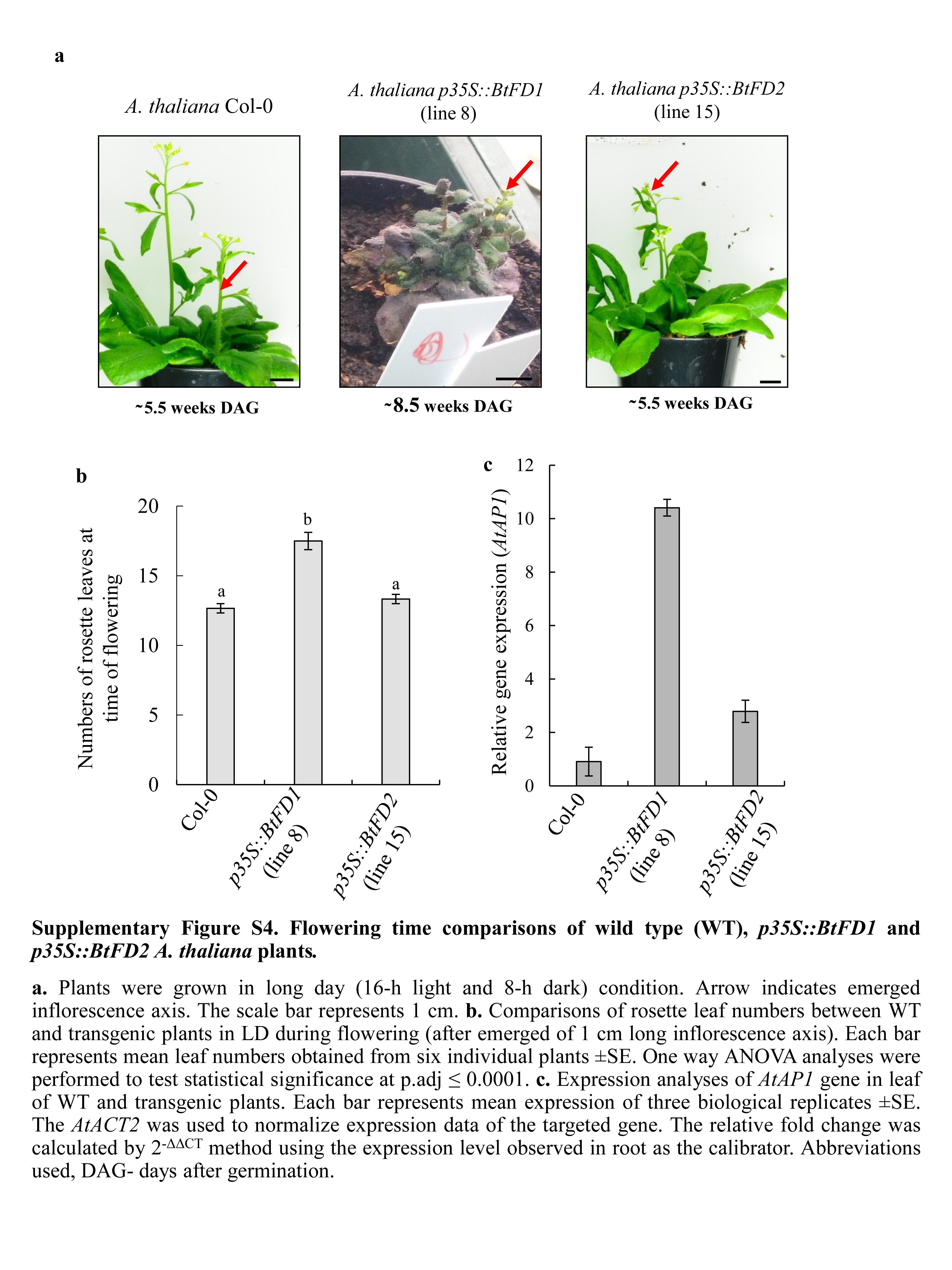

Supplement: Supplementary file 4 — Supplementary Information 4. [file 41598_2021_87491_MOESM4_ESM.tif]

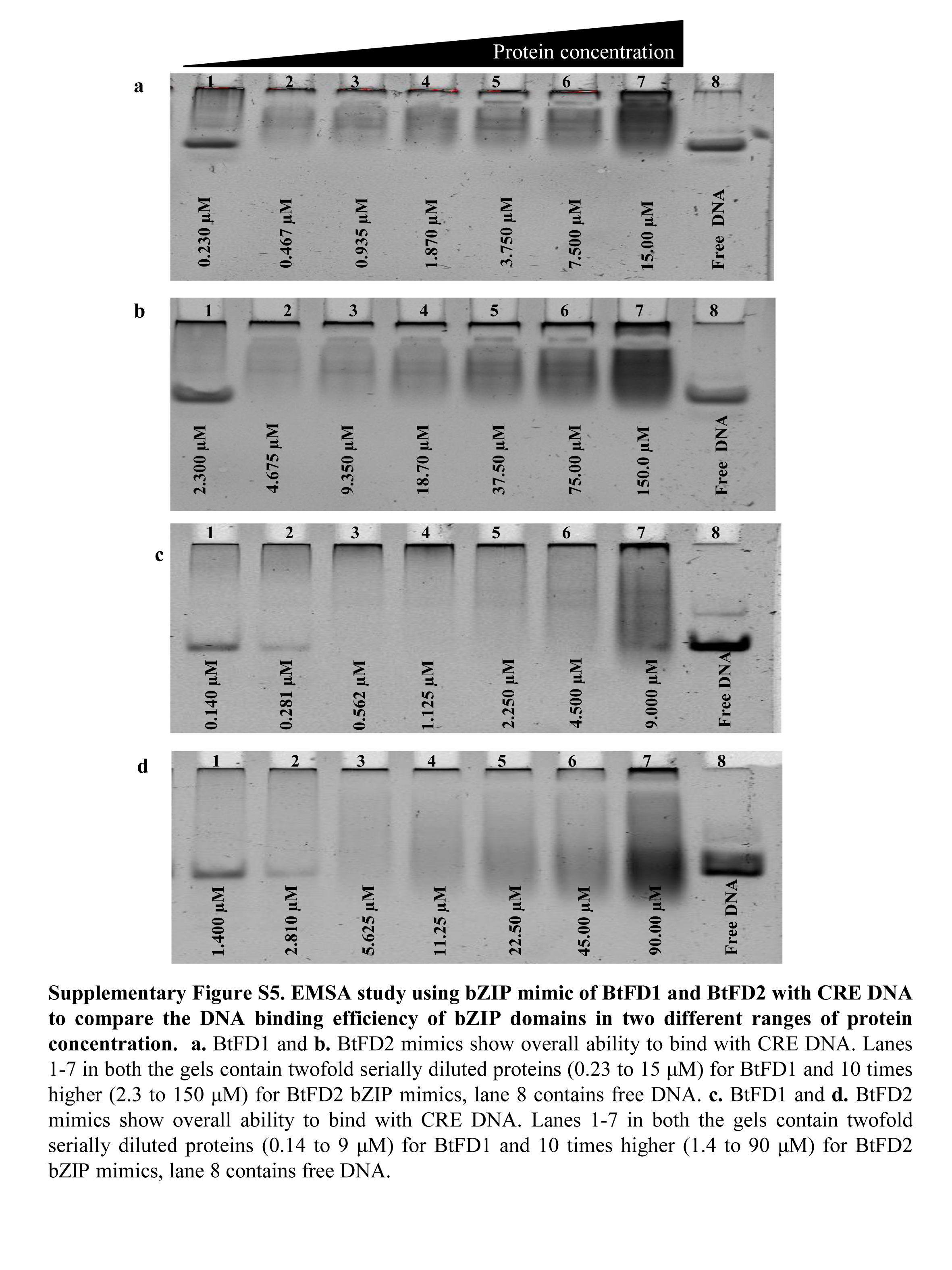

Supplement: Supplementary file 5 — Supplementary Information 5. [file 41598_2021_87491_MOESM5_ESM.tif]
