# Supplementary material for: Identification and functional characterization of two bamboo FD gene homologs having contrasting effects on shoot growth and flowering
Source: Sci Rep. 2021 Apr 12;11:7849. doi: 10.1038/s41598-021-87491-6 (PMC8041875; doi:10.1038/s41598-021-87491-6)
Supplement: Supplementary file 6 — Supplementary Information 6. [file 41598_2021_87491_MOESM6_ESM.docx]

**Supplementary Table S1. Summary of primers designed for real time RT-qPCR and PCR experiments.**

| **Target genes** | **Oligo names** | **Sequences (5’-3’)** | **Purposes** |
| --- | --- | --- | --- |
| *BtFD1* | BtFD1_QRT_F | GCAGGAAAACGAGAAGCTCCGTGTCA | Gene expression analyses by real time RT-qPCR |
|  | BtFD1_QRT_R | TCAGAATGGCGCCGAGAGC |  |
| *BtFD2* | BtFD2_QRT_F | ATGGCGGCCAACTACCACCACT |  |
|  | BtFD2_QRT_R | CGGTTCCTCATCATCCTGATGG |  |
| *BteIF4α* | BteIF4α_F | ATGCTCTCCCGTGGTTTCAAG |  |
|  | BteIF4α_R | CAAGGGTAAGCTCATCTCTCTTCAC |  |
| *BtFD1* | BtFD1_GW_F | ggggacaagtttgtacaaaaaagcaggcttaATGGCCATGGAGGACGAC | PCR amplification for Gateway cloning |
|  | BtFD1_GW_R | ggggaccactttgtacaagaaagctgggTCAGAATGGCGCCGAGAG |  |
| *BtFD2* | BtFD2_GW_F | ggggacaagtttgtacaaaaaagcaggcttaATGGCGGCCAACTACCAC |  |
|  | BtFD2_GW_R | ggggaccactttgtacaagaaagctgggTCAGAATTGCGTCGATGAGG |  |
| *BtFD1* | BtFD1_F | ATGGCCATGGAGGACGACG | Molecular confirmation of transgenic plants |
| *BtFD2* | BtFD2_R | TCAGAATTGCGTYGATGAGGTTC |  |
| *AtAP1* | AtAP1_F | ACCAAATCCAGCATCCTTAC | Gene expression analyses by real time RT-qPCR |
|  | AtAP1_R | AGTTCGAGATCATTCCTCCTC |  |

| *AtACT2* | AtACT2_F | AGTGGTCGTACAACCGGTATTGT |  |
| --- | --- | --- | --- |
|  | AtACT2_R | GATGGCATGAGGAAGAGAGAAAC |  |
